# Quantum–Classical Approach for Calculations of Absorption and Fluorescence: Principles and Applications

**DOI:** 10.1021/acs.jctc.1c00777

**Published:** 2021-10-07

**Authors:** Yakov Braver, Leonas Valkunas, Andrius Gelzinis

**Affiliations:** † Institute of Chemical Physics, Faculty of Physics, Vilnius University, Saulėtekio Avenue 9-III, LT-10222 Vilnius, Lithuania; ‡ Department of Molecular Compound Physics, Center for Physical Sciences and Technology, Saulėtekio Avenue 3, LT-10257 Vilnius, Lithuania

## Abstract

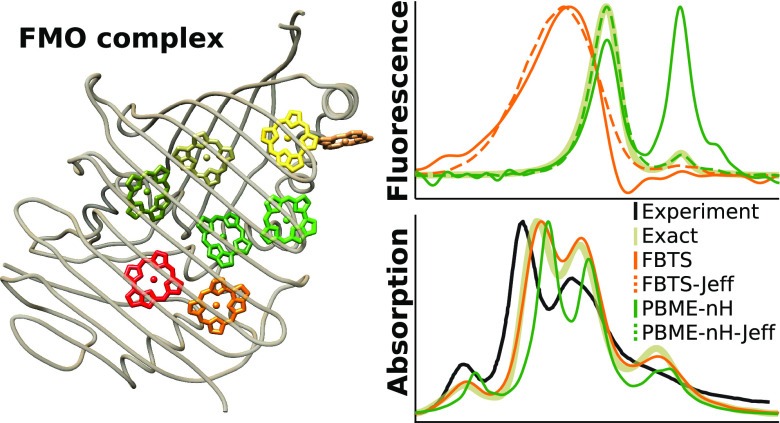

Absorption and fluorescence spectroscopy techniques provide a wealth of information on molecular systems. The simulations of such experiments remain challenging, however, despite the efforts put into developing the underlying theory. An attractive method of simulating the behavior of molecular systems is provided by the quantum–classical theory—it enables one to keep track of the state of the bath explicitly, which is needed for accurate calculations of fluorescence spectra. Unfortunately, until now there have been relatively few works that apply quantum–classical methods for modeling spectroscopic data. In this work, we seek to provide a framework for the calculations of absorption and fluorescence lineshapes of molecular systems using the methods based on the quantum–classical Liouville equation, namely, the forward–backward trajectory solution (FBTS) and the non-Hamiltonian variant of the Poisson bracket mapping equation (PBME-nH). We perform calculations on a molecular dimer and the photosynthetic Fenna–Matthews–Olson complex. We find that in the case of absorption, the FBTS outperforms PBME-nH, consistently yielding highly accurate results. We next demonstrate that for fluorescence calculations, the method of choice is a hybrid approach, which we call PBME-nH-Jeff, that utilizes the effective coupling theory [GelzinisA.;J. Chem. Phys.2020, 152, 0511033203545510.1063/1.5141519] to estimate the excited state equilibrium density operator. Thus, we find that FBTS and PBME-nH-Jeff are excellent candidates for simulating, respectively, absorption and fluorescence spectra of real molecular systems.

## I Introduction

Spectroscopy experiments remain the most valuable tool for investigating the properties of molecular systems.^1−4^ From the experimental point of view, perhaps the most straightforward are the absorption and fluorescence measurements, but theoretical simulation of the outcomes of these experiments, especially fluorescence, is a challenging task. Several formally exact methods have been developed to calculate the absorption and fluorescence lineshapes: the hierarchical equations of motion (HEOM) approach^5,6^ and the stochastic path integral (SPI) method.^7^ However, these methods require an impractically large amount of computational resources for simulations of larger molecular systems, implying the need for simpler and faster, albeit approximate, schemes. By far the most widely used approximate approaches are based on the quantum master equation (QME) and the cumulant expansion.^1,3,8−15^ Only recently the calculation of optical lineshapes has been approached using completely different methods—the time-dependent variational approach,^16,17^ the density matrix renormalization group algorithm,^18^ the reaction-coordinate master equation,^19^ and the quantum–classical theory.^20^

The main advantage of the quantum–classical approaches is that they constitute a complete simulation tool, in principle being applicable to any system observable. In addition, the quantum–classical theory explicitly accounts for the state of the bath, which has to be done to simulate fluorescence or nonlinear spectroscopic experiments. This is contrary to QME-based approaches, where accounting for the effects of entanglement between the system and the bath is problematic.^21,22^ Indeed, current efforts to apply the QME to nonlinear spectra have to circumvent this issue, e.g., using the frozen modes scheme for the slow bath degrees of freedom.^15^ All this implies that the quantum–classical theory holds great promise for cases when different spectroscopic experiments (absorption, fluorescence, etc.) of a particular system have to be simulated at the same or similar theoretical level to extract model parameters. However, the quantum–classical theory has only been applied to calculations of the fluorescence lineshapes in its simplest form—using the mean-field framework^23,24^ or the surface hopping method.^25^ In search of a method that is more accurate than the mean-field theory and is less computationally demanding than the surface hopping scheme, we adapt the methods based on the quantum–classical Liouville equation^3,26^ (QCLE). Our goal is twofold. First, we provide a theoretical framework for calculations of both absorption and fluorescence spectra for the QCLE-based approaches. Second, we investigate the accuracy of such methods to provide definite recommendations regarding their applicability for real molecular systems.

In the first part of this paper, we apply the forward–backward trajectory solution^27^ (FBTS) and the non-Hamiltonian variant of the Poisson bracket mapping equation^28^ (PBME-nH) to the calculation of absorption lineshapes of molecular aggregates. We choose two systems for benchmarking—a molecular dimer and the well-known Fenna–Matthews–Olson (FMO) photosynthetic complex, which is often used as a benchmark system for comparison of theoretical approaches. We base our calculations of FMO on a recently proposed structure-based model.^29^ To obtain formally exact results for comparison, we rely on the aforementioned HEOM approach^5^ and the SPI method.^7^ We find that the FBTS and PBME-nH yield very similar lineshapes of excellent accuracy as long as a dimer is considered. Calculations of the FMO complex, on the other hand, clearly indicate that the FBTS is more accurate than PBME-nH for large systems.

The next part of this work presents the calculations of fluorescence lineshapes, again for a dimer and the FMO complex. We propose two hybrid approaches, which we call FBTS-Jeff and PBME-nH-Jeff, that utilize the effective coupling theory^30^ to estimate the excited state equilibrium density matrix required for fluorescence calculations. The results imply that for calculations of fluorescence, the method of choice is PBME-nH-Jeff as it yields accurate results while remaining effective in terms of computational time. All of the methods performed quite well when calculating a dimer, but in the case of the FMO complex, the FBTS and FBTS-Jeff lead to qualitatively incorrect lineshapes. Meanwhile, PBME-nH tuned out to be applicable only in the high-temperature regime (*T* = 300 K), and it required two orders of magnitudes more computational time than PBME-nH-Jeff.

We therefore propose that for deep investigations of a particular system, a combination of FBTS for absorption and PBME-nH-Jeff for fluorescence is recommended.

## II Theory

### II.I Definition of the System

In this work, we consider the Frenkel exciton model^31^ for an electronic subsystem coupled to a harmonic bath with the following Hamiltonian
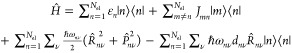
1The first line corresponds to the subsystem Hamiltonian *Ĥ*_S_, while the sums in the second line are the bath Hamiltonian *Ĥ*_B_ and the subsystem–bath interaction Hamiltonian *Ĥ*_SB_. Further, *N*_el_ is the number of electronic states in the subsystem, |*n*⟩ denotes the state where only the *n*th site is excited, ε_*n*_ is the corresponding excitation energy, and *J*_*mn*_ is the Coulombic resonance coupling between the corresponding excited states. The bath oscillators are assumed to be uncorrelated, and the frequency of the *v*th oscillator interacting with the *n*th electronic level is denoted by ω_*nν*_. The dimensionless coordinate and momentum operators of the oscillators are denoted by *R̂*_*nν*_ and *P̂*_*nν*_, respectively, and the double sums are over all oscillators and electronic levels. Finally, parameter *d*_*nν*_ is the dimensionless system–bath coupling constant determined from the spectral densities
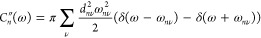
2The total system–bath coupling strength is measured by the reorganization energies
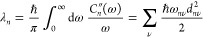
3We used two different models for the spectral density in our calculations, which, for simplicity, is taken to be the same for all the sites. First, the Debye spectral density^1,3^
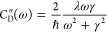
4which is often employed for calculations due to the resulting exponential correlation function;^12,19,32−36^ here, γ^–1^ is the bath relaxation timescale. Second, we used the B777 spectral density that was obtained from the fits of the fluorescence line-narrowing spectrum of the bacteriochlorophyll B777 complex^8^
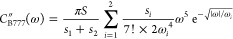
5with
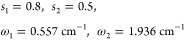
6Here, *S* is the Huang–Rhys factor that has to be chosen according to the system under consideration. It is related to the reorganization energy by . This spectral density has been widely employed by Renger and co-workers for studies on a number of photosynthetic complexes.^37−40^

### II.II Quantum–Classical Methods

Combining the methods of quantum and classical dynamics may be approached in several ways, and one of them is based on the quantum–classical Liouville equation^3,26^ (QCLE)
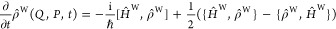
7Here, {·,·} denotes the Poisson brackets, and the index “W” refers to the partial Wigner transform^3,26^ of the corresponding quantity with respect to the bath degrees of freedom. The transformed density matrix ρ̂^W^(*Q*, *P*, *t*) and the Hamiltonian *Ĥ*^W^(*Q*, *P*) act as operators only in the subsystem Hilbert space. Their elements become functions of the bath phase-space variables—the set of coordinates *Q* and momenta *P* of the bath oscillators. It is important to note that the QCLE is exact in the case of a harmonic bath that interacts linearly with the subsystem degrees of freedom, as in eq 1.

A large number of bath degrees of freedom required to simulate a realistic environment precludes solving the QCLE directly, therefore, an approximate approach is needed. The first such approach adopted in the present work is the forward–backward trajectory solution^27^ (FBTS) proposed by Hsieh and Kapral. The FBTS is essentially a path integral-like solution of the QCLE that utilizes the mapping basis^41−43^ to describe the subsystem in terms of fictitious harmonic oscillators with well-defined coordinates *q* and momenta *p*. Working in the mapping basis allows one to calculate the (infinite number of) intermediate integrals analytically and obtain the following result^27^ (here given for an arbitrary operator *Â*^W^)
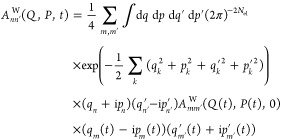
8Here, *A*_*mm*′_^W^(*Q*(*t*), *P*(*t*), 0) is the element (*m*, *m*′) of the initial operator *Â*^W^ where the bath coordinates and momenta are taken at time *t*. We note that the derivation of the FBTS requires effectively doubling the subsystem Hilbert space so that each subsystem state is described by a pair of fictitious oscillators whose phase-space variables are (*q*, *p*) and (*q*′, *p*′). The evolution of the subsystem and bath phase-space coordinates is calculated using a set of Hamilton’s equations that are classical in nature and scale linearly with increasing bath size. We refer the reader to the original works^27,44,45^ for the detailed derivation and analysis of FBTS.

Another quantum–classical method applied in our study is a variant of the Poisson bracket mapping equation^28,46−48^ (PBME). This method bears a close similarity to the FBTS as its derivation yields an expression for the evolution of a partially Wigner-transformed operator similar to the FBTS formula (eq 8). In the PBME framework, however, each subsystem state is mapped to only a single fictitious oscillator. An improvement of the original version^46^ of PBME has been proposed in ref (28), whereby Hamilton’s equations for the evolution of the phase-space variables are complemented with an additional term. This modified approach, called PBME-nH, has been shown to provide more accurate results when calculating the system dynamics than the original PBME in all of the tested cases.^28^ The FBTS is also more accurate than the PBME in most of the cases as demonstrated in our previous work.^49^ We therefore applied the PBME-nH variant for calculating the optical spectra, and the original PBME variant was not considered.

### II.III Calculation of Absorption Spectra

According to the response function theory, the absorption lineshape of a system is given by^1,3^
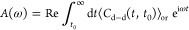
9Here, we omit the dimensional prefactor of ω/2ℏϵ_0_*cn*_r_, where ϵ_0_ is the electric permittivity of vacuum, *c* is the speed of light, and *n*_r_ is the refractive index of the sample. Further, ⟨·⟩_or_ denotes the orientational averaging (we assume an ensemble of molecules randomly oriented with respect to the polarization of the incident light), and *C*_d–d_(*t*, *t*_0_) is the dipole–dipole correlation function
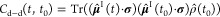
10Here, **σ** is the polarization of the incoming light pulse, and **μ̂**^I^(*t*) is the dipole moment operator in the interaction picture
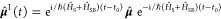
11The dipole moment operator describes the optical coupling between the excited and the ground states, and it may be expressed as
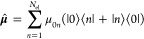
12where we assume μ_0*n*_ = μ_*n*0_. The time evolution of this operator may be calculated by directly applying the FBTS or PBME-nH formula.

The initial conditions for our simulations were chosen such that the subsystem is uncorrelated to the bath at *t* = *t*_0_, when the absorption experiment starts. Therefore, the initial density matrix may be factorized into subsystem and bath parts
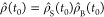
13with the same factorization holding for the initial partially Wigner-transformed density matrix. The subsystem is taken to occupy the ground state at *t* = *t*_0_, which may be expressed as ρ_00_(*t*_0_) = 1 and ρ_*mn*_(*t*_0_) = 0 for *m*, *n* = 1, ..., *N*_el_. The bath oscillators are taken to be in thermal equilibrium initially, so that the canonical energy distribution may be assumed, resulting in
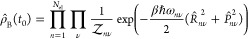
14where  is the partition function for the oscillator with frequency ω_*nν*_ and β = 1/(*k*_B_*T*). Carrying out the Wigner transformation, one arrives at the expression^1^
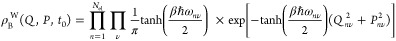
15In the quantum–classical framework, eq 10 is given by
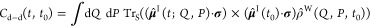
16Here, the total trace with respect to the bath and the subsystem is split into a trace with respect to the subsystem space (denoted by Tr_S_) and integration over the bath phase-space variables. We note that the FBTS and PBME-nH expressions for **μ̂**^I^(*t*; *Q*, *P*) contain an integration over the subsystem variables *q*, *p*, *q*′, and *p*′ (see eq 8); this integration may be performed together with the integration over *Q* and *P* in eq 16 by means of Monte Carlo (MC) sampling.

### II.IV Calculation of Fluorescence Spectra

The formula for the stationary fluorescence lineshape may be rigorously derived from the perspective of quantum electrodynamics,^1,50^ and the resulting expression is very similar to eq 9 with the exception of a different sign in the exponent (neglecting the dimensional prefactor of ω^3^/3π^2^ℏϵ_0_*c*^3^)
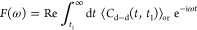
17Parameter *t*_1_ appearing in the correlation function denotes the moment the system has reached the excited state equilibrium after being excited with an infinitely short laser pulse at some *t* = *t*_0_ < *t*_1_. Calculation of the fluorescence spectrum thus requires calculating the excited state equilibrium density operator ρ̂^W^(*Q*, *P*, *t*_1_) (see eq 16). However, direct calculation of this quantity using FBTS or PBME-nH turns out to be inefficient due to slow convergence.^49^ This issue may be circumvented by means of an approximation: We may assume that the system–bath correlations may be neglected in the excited state so that eq 13 holds at *t* = *t*_1_ as well. Under this assumption, we have
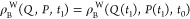
18that is, we identify the bath density matrix at time *t*_1_ with the distribution of bath coordinates and momenta propagated using Hamilton’s equations to time *t*_1_. Meanwhile, the equilibrium subsystem density matrix may be calculated by propagating projectors |*m*⟩⟨*n*| since
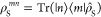
19The projectors may be propagated efficiently using FBTS or PBME-nH because they are pure subsystem operators, and no convergence issues arise.^49^ The dipole–dipole correlation function is therefore calculated using the formula
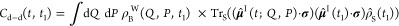
20where we used the fact that ρ̂_S_^W^(*t*_1_) ≡ ρ̂_S_(*t*_1_). We note that **σ** is understood here as the polarization vector of the emitted light.

It should be noted that factorization of the full density matrix to a subsystem and bath parts at *t* = *t*_1_ may be inadequate for strongly delocalized systems—such as the bacterial light-harvesting system^31^ (LH2)—as has been demonstrated in ref (12). On the other hand, the quantum–classical framework allows one to evolve the bath density operator until the excited state equilibrium is reached. Consequently, the subsystem–bath interaction effects are incorporated into ρ_B_^W^(*Q*, *P*, *t*_1_), which is not the case if we simply assume a canonical distribution for the bath, ρ̂_B_(*t*_1_) ∝ e^–β*Ĥ*_B_^.

Let us summarize the algorithm for calculating the fluorescence spectra using FBTS or PBME-nH. First, we generate a set of variables *q*, *p*, *q*′, *p*′, *Q*, and *P* and propagate the subsystem projector operators until equilibrium is reached to obtain ρ̂_S_(*t*_1_). Since the bath oscillators are propagated simultaneously with the subsystem variables, the bath density matrix ρ_B_^W^(*Q*, *P*, *t*_1_) is obtained in the process as well. The propagated bath coordinates *Q*(*t*_1_) and *P*(*t*_1_) are then kept, whereas the values of the subsystem variables *q*(*t*_1_), *p*(*t*_1_), *q*′(*t*_1_), and *p*′(*t*_1_) are discarded, and a new set is generated to prepare for the calculation of the dipole moment operator. Next, we propagate the dipole moment operator for a set period of time that ensures that the correlation function will have decayed considerably by the end of this period. According to the MC integration method, all of the described operations have to be repeated numerous times with different initial conditions, and the results should be averaged. Having obtained the averaged dipole–dipole correlation function, it only remains to perform its Fourier transform as given by eq 17 (note that in an actual calculation we may conveniently set *t*_1_ = 0). A detailed description of this algorithm may be found in the Supporting Information.

Finally, we note that the orientational averaging present in eqs 9 and 17 may be performed analytically if we assume that all orientations of the dipole moments with respect to the polarization are equally probable. The result is just a constant factor^51^
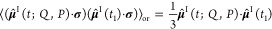
21The source code of the package that we wrote to perform the calculations is available on Gitlab.^52^

### II.V Application of the Effective Coupling Theory

The FBTS and PBME-nH are approximate methods, and depending on the system parameters they may yield the equilibrium system density matrix with high error.^28,44,49^ The error in the calculation of the fluorescence spectra may possibly be reduced if we find a more accurate way of estimating the value of ρ̂_S_(*t*_1_), as was suggested in ref (53). The equilibrium state of a molecular system has been investigated in ref (30), where it is shown that, to a good approximation, the equilibrium subsystem density operator is given by the familiar Boltzmann distribution
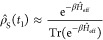
22calculated using the following effective Hamiltonian
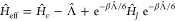
23where *Ĥ*_ε_ and *Ĥ*_*J*_ are, respectively, the diagonal and off-diagonal parts of *Ĥ*_S_, and Λ̂ = diag({λ_*n*_}). As we can see, the influence of the bath comes down to a change of the resonance couplings between subsystem states. Even though formula 23 presents an approximation that breaks down in the limits of low temperatures, fast baths, or very strong subsystem–bath interaction strengths,^30^ its range of applicability is a rather broad one. In all of the regimes of a dimer model studied below, this effective coupling theory predicted the values of the elements of ρ̂_S_(*t*_1_) with an error of less than 5% of the exact value (calculated using HEOM). We will refer to the basis in which *Ĥ*_eff_ is diagonal as the global basis (GB).

We may thus come up with the hybrid approaches, which we will refer to as FBTS-Jeff and PBME-nH-Jeff, whereby the general algorithm remains largely the same, but ρ̂_S_(*t*_1_) is calculated using eq 23 rather than FBTS of PBME-nH. Note that we nevertheless have to propagate Hamilton’s equations until the excited state equilibrium is reached to obtain ρ_B_^W^(*Q*, *P*, *t*_1_) (see eq 18). Further testing of these hybrid approaches has also revealed that the most accurate results are obtained if the subsystem phase-space variables are not discarded after the equilibrium is reached, but are kept and continued to be propagated in the second phase of the algorithm, when the dipole moment operator is being calculated. The suggestion of such hybrid approaches is one of the key novelties of the present work.

## III Results

In this section, we present the results of calculations of absorption and fluorescence spectra obtained using the methods introduced in the previous section. We have thoroughly investigated the accuracy of these methods in the case of a molecular dimer. We chose a set of “default” parameters of a dimer
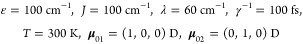
24and calculated the spectra varying one of the parameters while keeping others fixed. In eq 24, we defined the energy gap ε ≡ ε_2_ – ε_1_, and the corresponding system Hamiltonian is thus
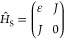
25The energy gap between the lowest excited state and the ground state was set to 15 000 cm^–1^, and it was accounted for by a corresponding shift of the final spectrum.

**Figure 1:**
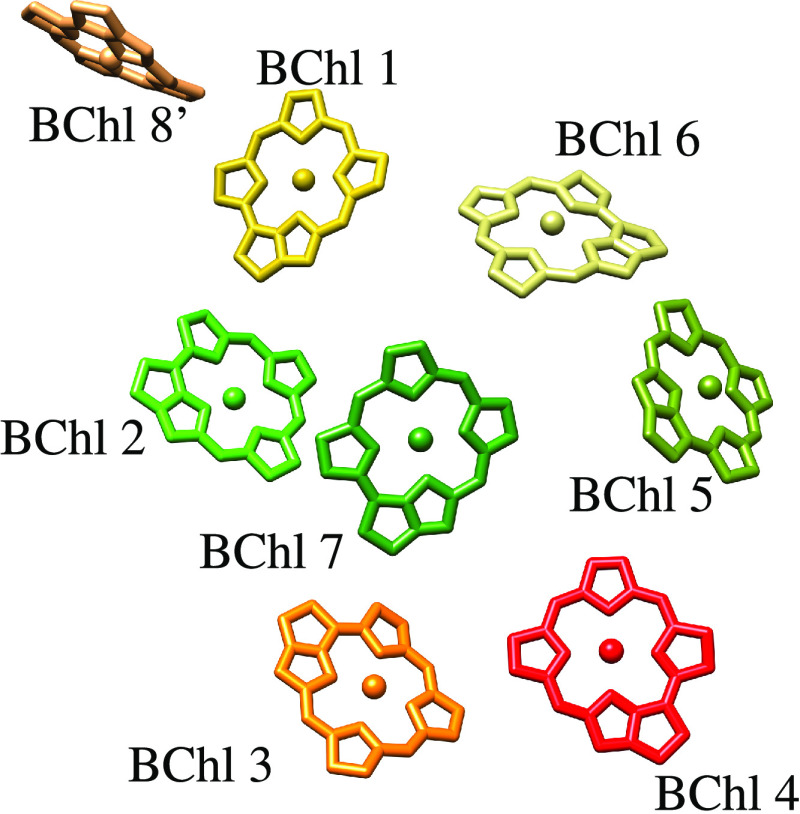
Pigment organization and numbering of the monomeric FMO complex of *P. aestuarii*. Structural data taken from the 3eoj PDB structure.^54^ The eighth BChl molecule is denoted with a prime because formally it belongs to another monomer in the FMO trimer. BChl molecules are depicted as porphyrins for clarity. The figure is created using UCSF Chimera.^55^

To investigate a more realistic example, we performed calculations of the FMO complex of *Prosthecochloris aestuarii* for a set of temperatures. The FMO complex, which is found as a trimer *in vivo*, acts as an energy conduit between the chlorosome and the reaction centers in the green sulfur bacteria.^56^ We have used a simplified structure-based model from ref (29) and considered FMO as a monomer, which has a pigment distribution shown in Figure 1. The energies of the pigments along with the standard deviations of the Gaussian energy distributions were taken from ref (29). The resonance couplings between the pigments were calculated as the charge interaction energy using atomic coordinates 3eoj PDB structure^54^ and the atomic transition charges given in ref (29). The excited state Hamiltonian elements are listed in Table 1. We have used the B777 spectral density (eq 5) with *S* = 0.5 as in ref (29). The transition dipole moments of BChl molecules, listed in Table 2, were calculated using atomic coordinates 3eoj PDB structure^54^ and the atomic transition charges given in ref (29). Following ref (29), in our calculations of absorption spectra we assumed that 65% of the FMO complexes present in the sample consist of seven BChl molecules, and only 35% of the complexes feature all eight BChls. Our results thus represent a weighted sum of such calculations.

**Table 1:** Excited State Hamiltonian Matrix Elements (in cm^–1^) and Standard Deviations of Energy Distributions (in cm^–1^) of the FMO Complex^a^

	BChl 1	BChl 2	BChl 3	BChl 4	BChl 5	BChl 6	BChl 7	BChl 8′	σ
BChl 1	12 650.70	–109.89	5.46	–6.12	7.10	–19.78	–8.10	26.47	36.9
BChl 2	–109.89	12 414.10	31.64	7.97	1.76	12.38	4.26	4.85	45.4
BChl 3	5.46	31.64	12 195.30	–67.30	–0.13	–9.26	–2.57	0.57	54.6
BChl 4	–6.12	7.97	–67.30	12 394.60	–69.58	–18.73	–63.21	–1.58	39.5
BChl 5	7.10	1.76	–0.13	–69.58	12 557.60	76.43	2.67	4.07	36.5
BChl 6	–19.78	12.38	–9.26	–18.73	76.43	12 527.90	31.82	–9.59	64.3
BChl 7	–8.10	4.26	–2.57	–63.21	2.67	31.82	12 478.50	–11.37	50.4
BChl 8′	26.47	4.85	0.57	–1.58	4.07	–9.59	–11.37	12 697.40	92.6

a See the text for details.

**Table 2:** Transition Dipole Moments of the FMO Complex in debye^a^

	μ_*x*_	μ_*y*_	μ_*z*_
BChl 1	–0.037	–1.536	5.279
BChl 2	4.157	–3.147	1.691
BChl 3	5.293	–0.421	–1.863
BChl 4	0.080	–2.253	5.015
BChl 5	4.182	–3.554	–0.282
BChl 6	–4.714	–2.081	2.005
BChl 7	–1.182	0.529	5.380
BChl 8′	–1.884	–5.163	–0.807

a See the text for details.

We used 10^6^ MC samples for the quantum–classical calculations unless noted otherwise, and the spectral density was cut off and discretized according to the conclusions of our previous analysis.^49^ Other methods that we used for comparison include the HEOM approach,^6^ which is formally exact for the Debye spectral density, eq 4, the approximate ctR method,^14^ which was shown to be both fast and accurate, and the SPI method,^7^ which allowed us to obtain formally exact absorption lineshapes for the FMO complex modeled using the B777 spectral density, eq 5. HEOM calculations were performed using a sufficient number of exponential terms in the expansion of the correlation function so that the accuracy criterion established in ref (58) is satisfied, while the depth of the equation hierarchy was being increased until convergence. Note that for calculations of absorption spectra, instead of ctR we could have used a spiritually similar full-cumulant expansion (FCE) method,^7,59^ which can account for coherence transfer in the system. This is an advantage of FCE over ctR (the latter being based on the cumulant expansion as well), although it comes at an increased numerical cost. In our dimer calculations, no coherence transfer is possible, however, due to perpendicular transition dipole moments. A brief comparison of the absorption spectra calculated using FCE and ctR is given in the Supporting Information.

### III.I Absorption

#### III.I.I Dimer

**Figure 2:**
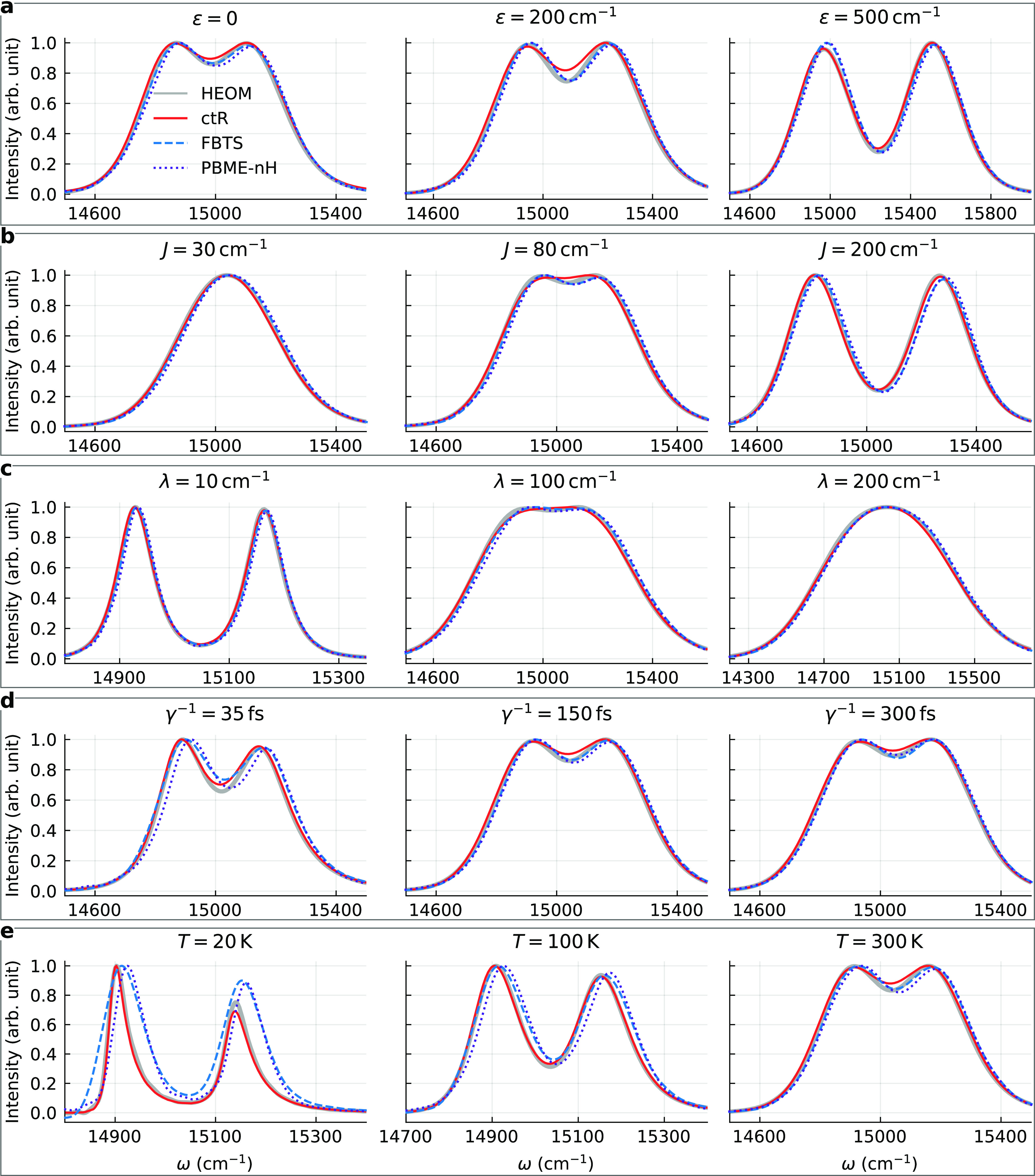
Absorption lineshapes of a family of dimers with different parameters. The lineshapes are normalized to unit maximum intensity.

Figure 2 shows the absorption spectra of a family of dimers calculated using the Debye spectral density and without energy disorder. Variation of the energy gap is displayed in Figure 2a. The FBTS and PBME-nH results are close to being identical, and they are highly accurate. For smaller energy gaps (ε = 0, 200 cm^–1^), the ctR approach is less accurate than the quantum–classical theory at estimating the intensity in the region around the saddle point.

Analysis of the different strengths of the resonance coupling and the coupling with the environment is provided in Figure 2b,c. There, the FBTS yields accurate results even for strong couplings, although the FBTS has been demonstrated to be inapplicable for calculations of system dynamics in these regimes.^49^ This may be attributed to the fact that the optical response function of a dimer decays within ∼200 fs in the mentioned cases, and at such short times, the quantum–classical theory remains reasonably accurate. The PBME-nH results are again very similar.

For “faster” baths (γ^–1^ = 35 fs), the quantum–classical theory is less accurate compared to when the relaxation time is greater (γ^–1^ = 300 fs), but qualitative agreement with the exact results is ensured in both cases (see Figure 2d). In the former case, the FBTS captures the positions of the peaks slightly better than the PBME-nH. For slow baths, both methods yield almost identical lineshapes, which appear to match the HEOM results more closely than those calculated using ctR. The temperature dependence of the accuracy is shown in Figure 2e. The quantum–classical methods yield rather accurate results even for *T* = 100 K, despite this low-temperature regime already being outside of the range of applicability of FBTS when calculating system dynamics.^49^ However, if the bath temperature is lowered to 20 K, the quantum–classical methods overestimate the widths of the spectral bands, and quantitative correctness is lost. On the other hand, the ctR approach leads to a different curve, which is in excellent agreement with the exact lineshape.

Overall we find that for the dimer system, the FBTS and PBME-nH yield almost indistinguishable results, and the accuracy of the quantum–classical methods is very high, on par with that of the ctR approach. Of all of the studied regimes, the quantum–classical theory leads to inaccurate results only in the low-temperature case.

#### III.I.II FMO Complex

**Figure 3:**
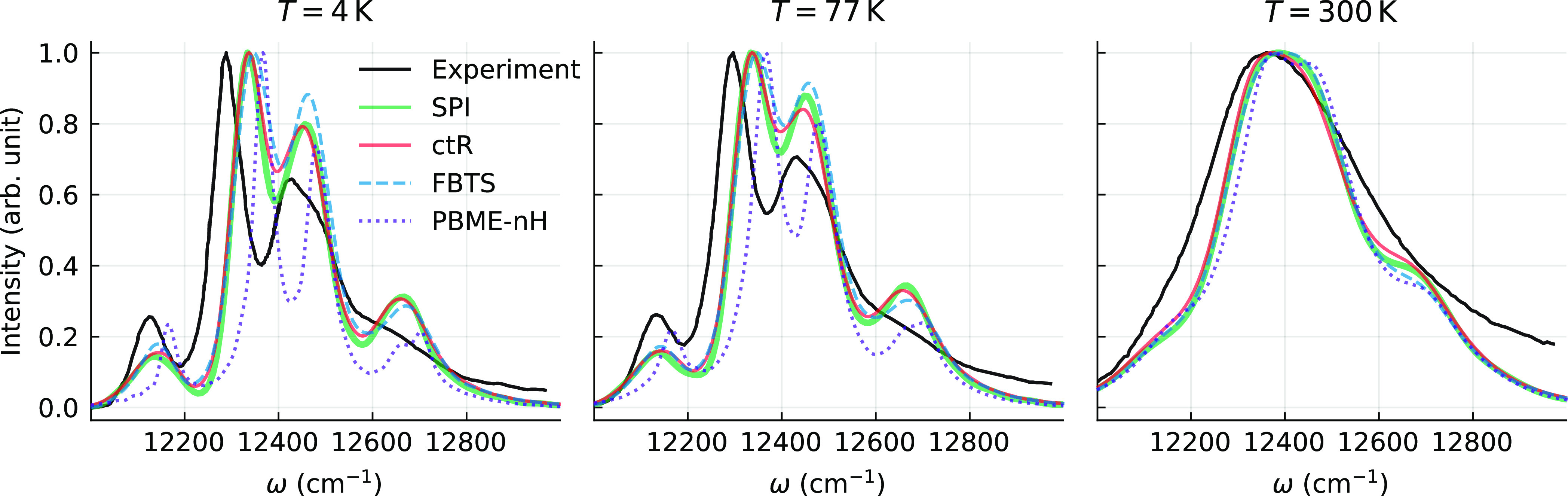
Absorption spectra of the FMO complex (see the text for details) at three different temperatures. The spectra are normalized to unit maximum intensity. Experimental spectra are taken from ref (57).

Calculated absorption spectra (which include the ω factor omitted in eq 9) of the FMO complex of *P. aestuarii* are shown in Figure 3 (the B777 spectral density was used, and energy disorder was taken into account). The experimental data provided in Figure 3 is taken from ref (57). As we can see, the qualitative agreement between the experimental data and the formally exact SPI results is reasonable at all temperatures. This is to be expected, having in mind that the parameters of the structure-based model that we employ^29^ have not been adjusted via a fitting procedure. We observe excellent agreement between the ctR and the exact stochastic path integral calculations. The FBTS also demonstrates results that are very close to the exact ones. On the other hand, the accuracy of PBME-nH is satisfactory only for *T* = 300 K, and the difference between the quantum–classical methods is more pronounced at lower temperatures, as was also observed when studying a dimer. The FBTS is therefore a better candidate among the quantum–classical methods for calculating the absorption spectra of real molecular systems.

### III.II Fluorescence

Now let us consider the fluorescence lineshapes. Our calculations have shown that the emission lineshapes calculated using PBME-nH and PBME-nH-Jeff methods are always blue-shifted by at least λ/2 compared to the exact results. Based on this observation, we argue that artificially red shifting the lineshapes obtained using these methods by λ/2 allows one to partially compensate for the approximate nature of these methods.

All of the results presented below were obtained using the Debye spectral density and without energy disorder. When calculating the spectra of the FMO complex, we used λ = 35 cm^–1^ and γ^–1^ = 100 fs.

#### III.II.I Dimer

**Figure 4:**
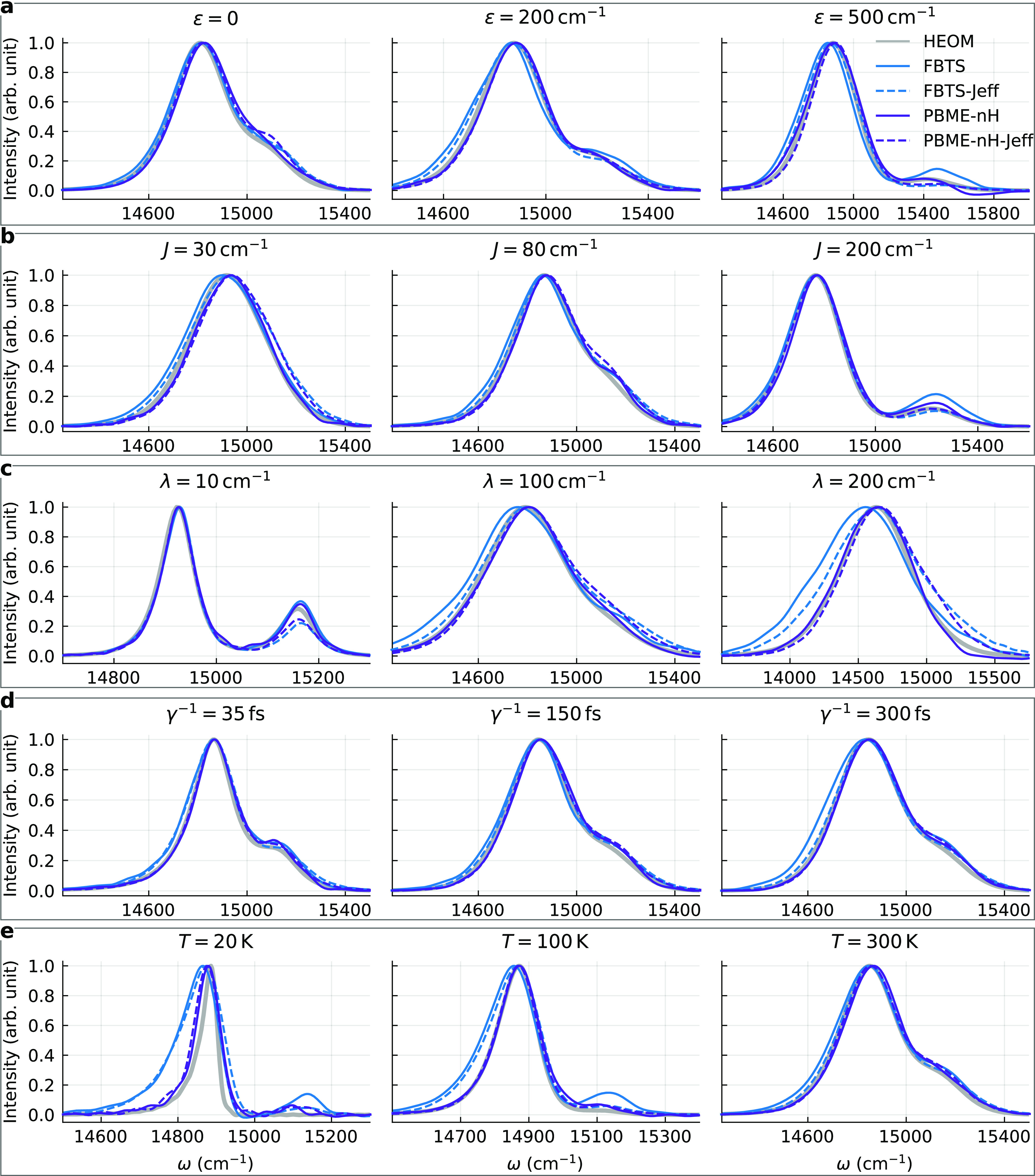
Fluorescence lineshapes of a family of dimers with different parameters. The lineshapes are normalized to unit maximum intensity.

The fluorescence spectra of dimers are depicted in Figure 4. As noted above, all of the PBME-nH and PBME-nH-Jeff emission lineshapes have been red-shifted by λ/2. Starting with the variation of parameter ε shown in Figure 4a, we indeed notice that the positions of the intensity maxima are predicted with a minimal error once the shift is applied. All of the quantum–classical methods yield similar results, although the FBTS is less accurate for larger energy gaps (ε = 200, 500 cm^–1^). In the case of ε = 200 cm^–1^, the PBME-nH gives the best results, but its error increases when ε = 500 cm^–1^. In the latter case, 10^8^ trajectories were required to reach fully converged (to visual accuracy) results, but a region of negative intensity around ε ≈ 15 700 cm^–1^ nevertheless remains. Note that two peaks can be observed in the presented fluorescence spectra as the thermal energy (*k*_B_*T*) is comparable to the energy gap in all cases.

Considering the variation of the resonance coupling strength shown in Figure 4b, all of the quantum–classical methods yield similar results that are rather accurate. The FBTS and FBTS-Jeff, however, overestimate the width of the spectral band in the weak resonance coupling regime (*J* = 30 cm^–1^). This deficiency is especially pronounced in the regime of a strong subsystem–bath interaction, λ = 200 cm^–1^ (see Figure 4c). One source of errors is the site-basis coherences of the equilibrium density operator—the equilibrium value of ρ_12_ calculated using FBTS is greater than the correct one by ∼30% at λ = 200 cm^–1^. Meanwhile, the PBME-nH predicts this element of the density matrix with an error of only ∼0.5%, which in turn allows one to obtain an accurate emission lineshape, although convergence was achieved using 10^7^ trajectories. The PBME-nH is the most accurate of the studied methods in the weak subsystem–bath coupling regimes as well.

Returning back to the default value of λ = 60 cm^–1^ and varying the bath relaxation time, we again notice that the spectra calculated using the quantum–classical methods hardly differ, as demonstrated in Figure 4d. The PBME-nH is once again better at estimating the width of the bands. However, the case of γ^–1^ = 150 fs required using 10^7^ trajectories. Similar results are seen in Figure 4e, where PBME-nH and PBME-nH-Jeff consistently outperform the FBTS-based methods at predicting the bandwidths, especially at lower temperatures. At *T* = 100 K, the FBTS overestimates the equilibrium value of ρ_12_ by ∼35% and that error causes inaccuracies in the final fluorescence lineshapes. On the other hand, PBME-nH and PBME-nH-Jeff yield reasonably accurate results even at *T* = 20 K. We note the FBTS and PBME-nH results corresponding to the cases *T* = 20 and 100 K were obtained using 10^7^ trajectories.

Overall, these calculations imply that for fluorescence, PBME-nH is more accurate than FBTS, contrary to the absorption calculations. Moreover, the use of the effective coupling theory prevents the appearance of nonphysical negative features in the lineshapes.

#### III.II.II FMO Complex

**Figure 5:**
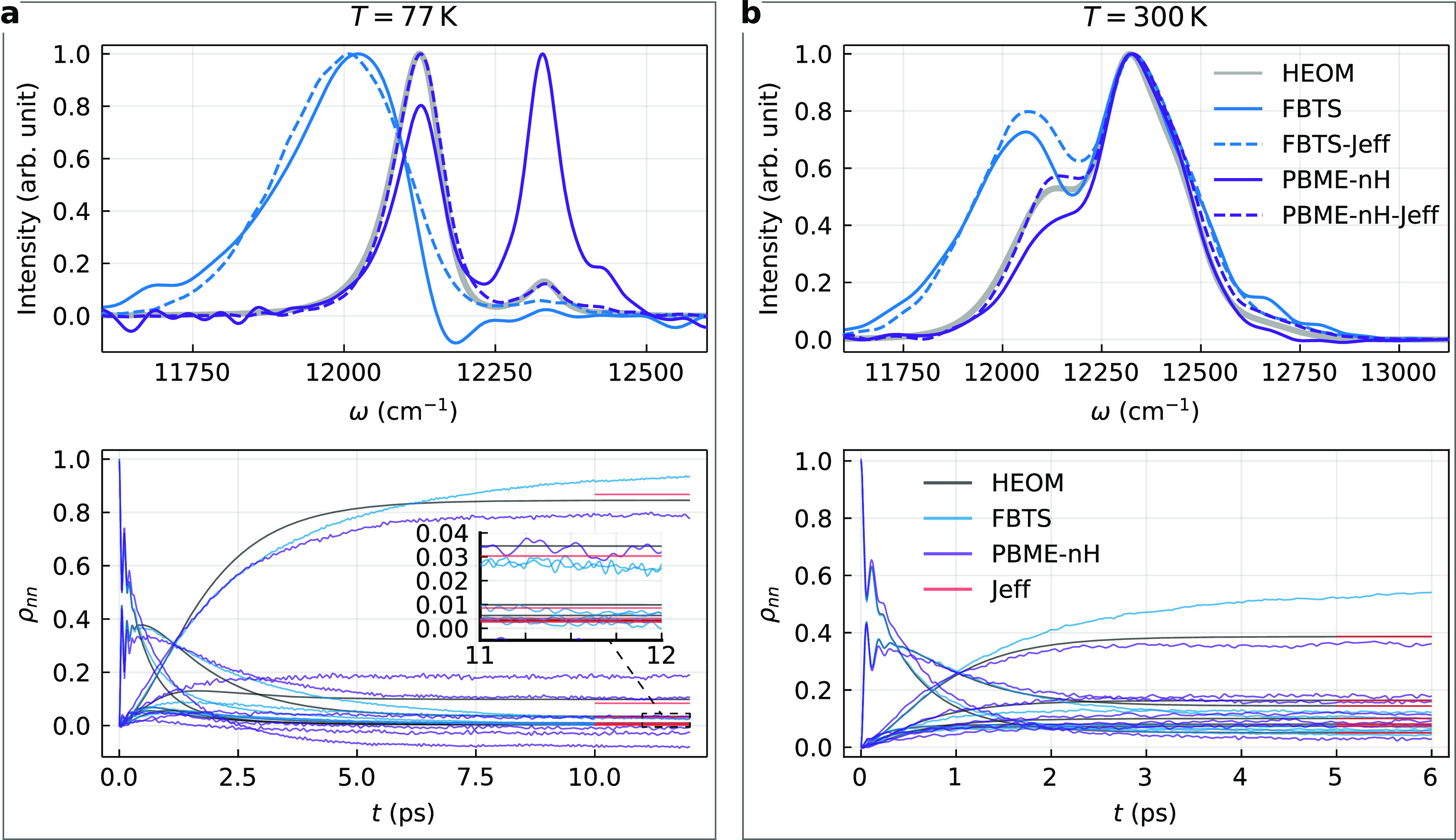
Fluorescence lineshapes of the seven BChls FMO complex (upper plots) and the site-basis population dynamics (lower plots) calculated using the Debye spectral density (λ = 35 cm^–1^, γ^–1^ = 100 fs) and no energy disorder at (a) *T* = 77 K, (b) *T* = 300 K. The spectra are normalized to unit maximum intensity. The *y*-coordinates of the red horizontal lines indicate the equilibrium populations as given by the effective coupling theory, eq 22.

Figure 5 shows the fluorescence lineshapes of the seven BChls FMO complex, and Figure 5a corresponds to the low-temperature case (*T* = 77 K). First, we notice that FBTS and PBME-nH results (obtained using 10^7^ trajectories) are inaccurate even at a qualitative level, which may be explained by the equilibrium density matrix being calculated with considerable error using these methods. This is illustrated in the lower plot in Figure 5a, where the evolutions of the populations of the site-basis energy levels are shown. The PBME-nH even predicts negative populations, which is a deficiency of this method that has been reported by its authors.^28^ This issue is attributed to the zero-point energy leakage that is a known issue of approximate quantum–classical methods,^28^ which is particularly pronounced at low temperatures. However, this is only a shortcoming of PBME-nH as an approximate solution of the QCLE, rather than the quantum–classical theory in general. An exact solution would yield correct equilibrium populations, and the detailed balance would not be violated. The accuracy of the effective coupling theory is also smaller for this seven-level system at *T* = 77 K (see the red lines in Figure 5a). Nonetheless, PBME-nH-Jeff yields an emission lineshape that closely matches the HEOM result. The FBTS-Jeff method, on the other hand, is not appreciably more accurate than the FBTS.

As we can see in Figure 5a, the HEOM fluorescence lineshape calculated using our model of the FMO complex features two emission bands, positioned at ε_1_ = 12 120 cm^–1^ and ε_2_ = 12 330 cm^–1^. Let us show that this is to be expected from the model that we employ. According to the effective coupling theory,^30^ the excited state equilibrium populations of the two lowest energy levels of the global basis (GB) are ρ_11_^GB^ = 0.95 and ρ_22_^GB^ = 0.026, while the corresponding magnitudes of the transition dipoles moments are |μ_01_^GB^|^2^ = 21 D^2^ and |μ_02_^GB^|^2^ = 54 D^2^. The intensity ratio of the two bands is thus on the order of ρ_11_^GB^|μ_01_^GB^|^2^/ρ_22_^GB^|μ_02_^GB^|^2^ = 14, which is consistent with the ratio of 7.6 observed in Figure 5a. It should be noted that the emission spectra of real FMO complexes are highly dependent on the type of organism they come from. For example, the fluorescence spectrum of the FMO protein of aerobic phototrophic acidobacterium Candidatus *Chloracidobacterium thermophilum* features only a single high-energy peak when measured at *T* = 77 K.^60^ Meanwhile, the experimental data corresponding to green photosynthetic bacterium *Chlorobium tepidum* indicates a more structured fluorescence spectrum at low temperatures.^61^

Turning to the room-temperature case (*T* = 300 K) shown in Figure 5b, the PBME-nH-Jeff again yields the most accurate results, and it requires only 10^6^ trajectories to obtain converged results. By contrast, PBME-nH requires two orders of magnitudes more trajectories to obtain the converged lineshape shown in the figure, but this method does not provide more accurate results. In the lower plot of Figure 5b, we can see that the effective coupling theory is highly accurate in this case and that PBME-nH is considerably more accurate at estimating the equilibrium density matrix (populations) than the FBTS. This explains the fact that the fluorescence spectrum calculated using FBTS is much less accurate compared to the PBME-nH result, with the former being quantitatively incorrect.

Additional calculations of the same system using the B777 spectral density yielded similar results to those discussed above (see the Supporting Information), leading to an analogous conclusion that the PBME-nH-Jeff method is the most appropriate for calculating the fluorescence spectra.

## IV Discussion and Conclusions

In this work, we have demonstrated that the studied quantum–classical methods, the FBTS and PBME-nH, may be successfully applied to calculations of optical lineshapes of molecular systems. For a two-site dimer system, both methods lead to almost identical absorption lineshapes, which are highly accurate on the quantitative level. Interestingly, essentially the same level of accuracy is achieved regardless of the values of the system parameters, although FBTS is slightly better at capturing the positions of the peaks for slow baths (γ^–1^ ∼ 35 cm^–1^) and at lower temperatures (*T* ∼ 100 K). However, in the case of a realistic system—the eight BChls FMO complex—the FBTS turned out to be considerably more accurate than the PBME-nH as the latter method predicted the positions of the bands, their relative intensities, and widths with noticeable error. The FBTS results, on the other hand, produced quantitatively correct results for this system even at very low temperatures (*T* = 4 K).

In the second part of this work, we considered the calculation of fluorescence lineshapes, which has not been approached using the quantum–classical Liouville equation before. Apart from directly applying the FBTS and PBME-nH, we combined these methods with the effective coupling theory^30^ that provides an accurate estimate of the excited state equilibrium density operator. The two resulting methods, FBTS-Jeff and PBME-nH-Jeff, differ from their original counterparts in that the equilibrium density matrix is calculated using the effective coupling theory rather than using the FBTS or PBME-nH. Additionally, we found an empirical rule that the fluorescence lineshapes calculated using PBME-nH and PBME-nH-Jeff should be artificially red-shifted by λ/2 for a more accurate result. As with the calculations of absorption lineshapes, all four methods yield similar results in the case of a dimer system. The dependence of the accuracy of the methods on the values of the system parameters is again not a pronounced one, although the FBTS-Jeff and especially the FBTS overestimate the widths of the bands when the system–bath coupling is strong (λ = 200 cm^–1^) or when the temperature is low (*T* ≲ 100 K). For a dimer, the most accurate emission lineshapes are those calculated using PBME-nH, but the PBME-nH-Jeff results are only slightly less accurate. However, the application of the effective coupling theory turned out to be especially beneficial when calculating fluorescence lineshapes of the FMO complex. At *T* = 77 K, the density matrix was calculated using FBTS and PBME-nH with substantial error, leading to qualitatively incorrect results. The FBTS-Jeff provided not much of an improvement over FBTS, but PBME-nH-Jeff allowed us to obtain the emission lineshape with excellent accuracy. At *T* = 300 K, the PBME-nH performed better than at *T* = 77 K in terms of both estimating the equilibrium density matrix and calculating the lineshape, but it required using 10^8^ MC samples. On the other hand, PBME-nH-Jeff lead to more accurate results with just 10^6^ MC samples. We therefore conclude that PBME-nH-Jeff is the best candidate for calculating the emission lineshapes of real molecular systems.

The results mentioned above also demonstrate the importance of benchmarking the computational methods on realistic systems for which formally exact results may be obtained. In the case of absorption, we have seen that the FBTS is almost identical to PBME-nH in terms of accuracy when studying a dimer, yet for a realistic system the FBTS turned out to be considerably more accurate. In the case of fluorescence, the PBME-nH seemed like the most suitable method as long as a dimer was considered, but calculations of the FMO complex clearly demonstrate that PBME-nH is actually not as robust as PBME-nH-Jeff. In the present work, we judged about the accuracy of the approximate methods by direct comparison with the exact results, but several additional accuracy criteria could be used, such as the oscillator strength sum rule^1^ or the detailed balance relation.^1,7^ The latter may in principle form a basis of a new computational methodology that would combine the imaginary-time formalism^7^ with the quantum–classical framework.

Finally, let us evaluate the amount of computational resources required to calculate the optical spectra using the methods that we found most useful. Utilizing 480 CPU cores of a high-performance computer, the calculation of the absorption spectrum of the seven BChls FMO complex at *T* = 4 K using FBTS with 10^6^ MC samples took ∼5 min. Naturally, obtaining the fluorescence lineshapes required more computational time since the system had to be first propagated until equilibrium is reached. Using PBME-nH-Jeff with 10^6^ MC samples, the emission lineshape of a seven BChls FMO complex at *T* = 77 K was obtained within ∼30 min. An important advantage of the MC integration scheme is that adding more integration dimensions does not increase the amount of samples required to reach convergence. In the present case, this allowed us to include energy disorder in the system while keeping the calculation time effectively unchanged. This is to be contrasted with the HEOM or ctR calculations, whereby the computational time increases linearly with the number of disorder realizations. The quantum–classical methods may therefore be used for fitting experimental data, which generally requires performing multiple program runs to find optimal parameters of the model. We also note that while the ctR theory allows one to calculate accurate absorption spectra more than 10 times faster than using the quantum–classical methods, the former does not allow one to calculate populations of the energy levels or fluorescence spectra. Therefore, it seems more natural to apply quantum–classical approaches when properties of a system beyond its absorption spectrum are of interest.

To summarize, in this paper we have provided the needed theoretical framework for calculations of absorption and fluorescence lineshapes using the QCLE-based approaches. Moreover, we have successfully incorporated the recently proposed effective coupling theory that allowed us to significantly increase the accuracy of fluorescence calculations with basically no additional computational effort. Even though the results obtained for a molecular dimer are very similar among all the considered methods, that is no longer the case for a real photosynthetic FMO complex. Our results show that the best accuracy is obtained using FBTS for absorption and PBME-nH-Jeff for fluorescence, and we therefore suggest this combination for future work.

## Supporting Information Available

Detailed description of the algorithm of calculation of fluorescence lineshapes, absorption lineshapes calculated using the full-cumulant expansion theory, and results of calculations of the fluorescence lineshapes of the FMO complex where the B777 spectral density was used (PDF)
