# Supplementary material for: Quantum–Classical Approach for Calculations of Absorption and Fluorescence: Principles and Applications
Source: J Chem Theory Comput. 2021 Oct 7;17(11):7157–68. doi: 10.1021/acs.jctc.1c00777 (PMC8719324; doi:10.1021/acs.jctc.1c00777)
Supplement: Supplementary file 1 — ct1c00777_si_001.pdf [file ct1c00777_si_001.pdf]

# Supporting Information for: "Quantum–Classical Approach for Calculations of Absorption and Fluorescence: Principles and Applications"

Yakov Braver,[1,2] Leonas Valkunas,[1,2] and Andrius Gelzinis[1,2,*]

[1]*Institute of Chemical Physics, Faculty of Physics, Vilnius University, Saulėtekio Ave. 9-III, LT-10222 Vilnius, Lithuania*
[2]*Department of Molecular Compound Physics, Center for Physical Sciences and Technology, Saulėtekio Ave. 3, LT-10257 Vilnius, Lithuania*

## I. ALGORITHMS FOR CALCULATION OF FLUORESCENCE LINESHAPES

In this section, we provide the detailed algorithms for calculating the fluorescence lineshapes using FBTS, FBTS-Jeff, PBME-nH, and PBME-nH-Jeff. Below, we use the notation $q \equiv \{q_1, \ldots, q_{N_{\text{el}}}\}$, $p \equiv \{p_1, \ldots, p_{N_{\text{el}}}\}$ (and similarly for $q'$ and $p'$), and $Q = \{Q_1, \ldots, Q_{N_{\text{el}} \times N_{\text{osc}}}\}$, $P = \{P_1, \ldots, P_{N_{\text{el}} \times N_{\text{osc}}}\}$, where $N_{\text{el}}$ is the number of excited states in the subsystem, and $N_{\text{osc}}$ is the number of oscillators used to discretize the spectral density.

The algorithm for FBTS and PBME-nH is the following:

1. Assuming the initial time moment, $t = t_0$, generate the initial conditions for the Hamilton's equations: Draw the values for the bath variables $Q(t_0)$, $P(t_0)$ from the Wigner-transformed canonical distribution, Eq. (15). For FBTS, generate the values for the subsystem variables $q(t_0)$, $p(t_0)$, $q'(t_0)$, $p'(t_0)$ from a $4N_{\text{el}}$-dimensional Gaussian distribution with zero mean and unit variance. For PBME-nH, generate the values for the subsystem variables $q(t_0)$, $p(t_0)$ from a $2N_{\text{el}}$-dimensional Gaussian distribution with zero mean and a variance of $1/\sqrt{2}$.

2. Propagate the Hamilton's equations for a certain time interval until equilibrium is reached (at some $t = t_1$) to obtain the values $q(t_1)$, $p(t_1)$, $q'(t_1)$, $p'(t_1)$, $Q(t_1)$, $P(t_1)$.

3. Apply the FBTS formula, Eq. (8), and a similar one for PBME-nH to calculate the projectors $\hat{A}_{mn} \equiv |m\rangle\langle n|$ in the Heisenberg picture at $t = t_1$. Denote the result by $\hat{A}_{mn}(t_1)$.

4. Calculate the system density matrix using the identity $\rho_{\text{S}}^{mn}(t_1) = \text{Tr}\big(\hat{A}_{mn}(t_1)\hat{\rho}_{\text{S}}(t_0)\big)$.

5. Generate a new set of values for the subsystem variables as in Step 1, and denote them by $q(t_1)$, $p(t_1)$, $q'(t_1)$, $p'(t_1)$.

6. Using the values $q(t_1)$, $p(t_1)$, $q'(t_1)$, $p'(t_1)$, $Q(t_1)$, $P(t_1)$ as the initial conditions, propagate the Hamilton's equations for a certain time interval until the dipole–dipole correlation function decays sufficiently (at some $t = t_2$). At each (discrete) propagation step in the range $t \in [t_1, t_2]$, save the values of the subsystem variables $q(t)$, $p(t)$, $q'(t)$, $p'(t)$.

7. Apply the FBTS formula, Eq. (8), and a similar one for PBME-nH to calculate the dipole moment operator $\hat{\boldsymbol{\mu}}^{\text{I}}\big(t; Q(t_1), P(t_1)\big)$ at each time moment in the range $t \in [t_1, t_2]$. Note that this operator depends on the values $Q(t_1)$, $P(t_1)$ parametrically. Indeed, in the quantum-classical framework, $\hat{\boldsymbol{\mu}}^{\text{I}}$ is calculated via the subsystem variables (see Eq. (8)) that, in turn, are connected to the bath variables through the Hamilton's equations.

8. According to the *Monte Carlo* (MC) integration scheme, calculate the $n$th sample of the dipole–dipole correlation function (Eq. (20)) at each time moment in the range $t \in [t_1, t_2]$ as

$$C_{\text{d-d}}^{n}(t, t_1) = \frac{1}{3}\text{Tr}_{\text{S}}\Big(\hat{\boldsymbol{\mu}}^{\text{I}}\big(t; Q(t_1), P(t_1)\big) \cdot \hat{\boldsymbol{\mu}}^{\text{I}}(t_1)\hat{\rho}_{\text{S}}(t_1)\Big). \tag{S1}$$

   Here, we used the result for the orientational averaging, Eq. (21). Note that the Wigner image of the excited state bath equilibrium density operator, $\rho_{\text{B}}^{\text{W}}(Q, P, t_1)$, from Eq. (20) is no longer present in Eq. (S1) because the equilibrium values $Q(t_1)$, $P(t_1)$ calculated at each iteration of the MC loop are distributed according to the $\rho_{\text{B}}^{\text{W}}(Q, P, t_1)$ function.

9. Repeat Steps 1–8 $N_{\text{MC}}$ times to obtain $N_{\text{MC}}$ samples of the dipole–dipole correlation function.

10. Average the obtained MC samples:

$$C_{\text{d-d}}(t, t_1) = \frac{1}{N_{\text{MC}}}\sum_{n=1}^{N_{\text{MC}}} C_{\text{d-d}}^{n}(t, t_1). \tag{S2}$$

   The resulting quantity, $C_{\text{d-d}}(t, t_1)$, is a discrete function in the range $t \in [t_1, t_2]$.

11. Assuming $t_1 = 0$ and $t_2 \to \infty$, calculate the final fluorescence lineshape using Eq. (17).

The algorithm for the hybrid approaches, FBTS-Jeff and PBME-nH-Jeff, is largely the same, except for the Steps 3–5. They are modified as follows:

3. Proceed to Step 4.

4. Calculate the subsystem density matrix, $\hat{\rho}_S(t_1)$, using the effective-coupling theory.[1] (This should actually be performed only once, prior to starting the MC loop, if no disorder is present)

5. Proceed to Step 6, where the values of the subsystem variables $q(t_1)$, $p(t_1)$, $q'(t_1)$, $p'(t_1)$ obtained in Step 2 will be used.

Our implementations of the above algorithms are available on Gitlab.[2]

## II. ABSORPTION LINESHAPES CALCULATED USING THE FULL-CUMULANT EXPANSION THEORY

In this section, we briefly compare the absorption lineshapes calculated using the ctR theory and the full-cumulant expansion[3] (FCE) approach. Figure S1 shows the absorption spectra of a dimer studied in the main text; three sample cases where the ctR results are not perfect are demonstrated. As we can see, the difference between the FCE and ctR results is insignificant. However, a more pronounced difference should be expected if coherence transfer takes place in the system since the possibility of such a process is totally neglected in the ctR theory.[3,4] The FCE theory, on the other hand, is capable of taking this effect into account, although this comes at an additional computational cost.[3]

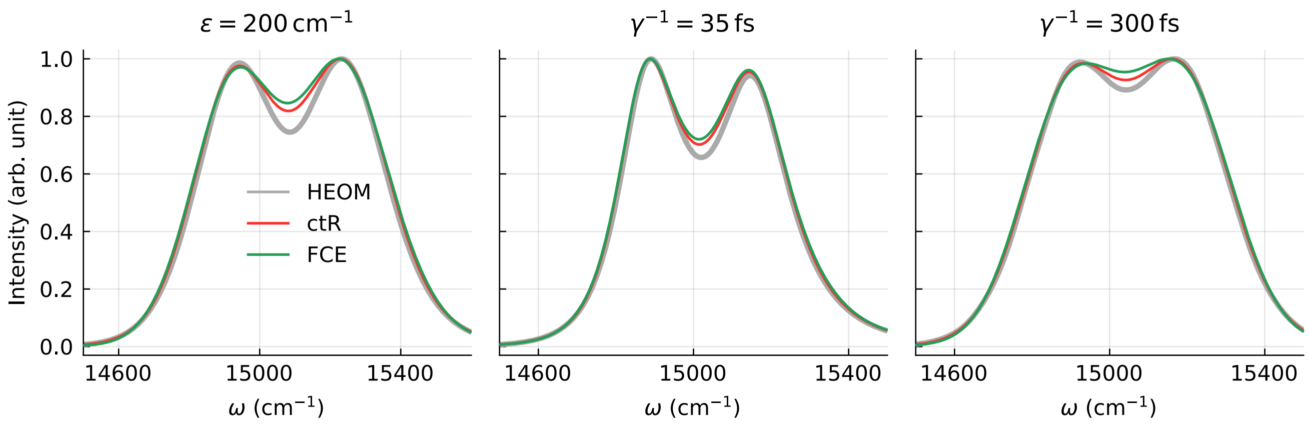


Figure S1. Absorption spectra of a dimer calculated using the ctR, FCE, and HEOM theories. The system parameters are taken from the default set analyzed in the main text. The parameter values specified in the figure override the corresponding default values.

## III. FLUORESCENCE SPECTRA OF THE FMO COMPLEX USING THE B777 SPECTRAL DENSITY

Figure S2 shows the fluorescence spectra of the 7 BChls FMO complex calculated using the B777 spectral density and no disorder. The FBTS and PBME-nH results were obtained using $10^8$ trajectories, while the FBTS-Jeff and PBME-nH-Jeff methods already yielded converged results with just $10^6$ trajectories. The obtained lineshapes are visually similar to those calculated using the Debye spectral density (shown in the main text), and similar conclusions regarding the accuracy of the quantum-classical methods can be drawn. In the low-temperature case (see Fig. S2a), the FBTS clearly leads to unphysical results since the spectrum features a region of negative intensity. The PBME-nH again predicts negative populations for some of the site-basis energy levels, hence the corresponding lineshape is likely to be incorrect as well. The PBME-nH-Jeff leads to a different curve, which has the intensities of the peaks at $\sim 12100\,\mathrm{cm}^{-1}$ and $\sim 12300\,\mathrm{cm}^{-1}$ differing by an order of magnitude. As described in the main text, this is consistent with the model of the FMO complex used in our calculations.

In the case of $T = 300\,\mathrm{K}$, the effective-coupling theory may be considered correct to within at least 1% at estimating the populations, therefore, we may conclude from the lower plot in Fig. S2 that the PBME-nH estimates the equilibrium populations with a smaller error than the FBTS. The corresponding PBME-nH lineshape should thus be closer to the correct one. As in the case of Debye spectral density, the PBME-nH-Jeff results are similar to those obtained using PBME-nH.

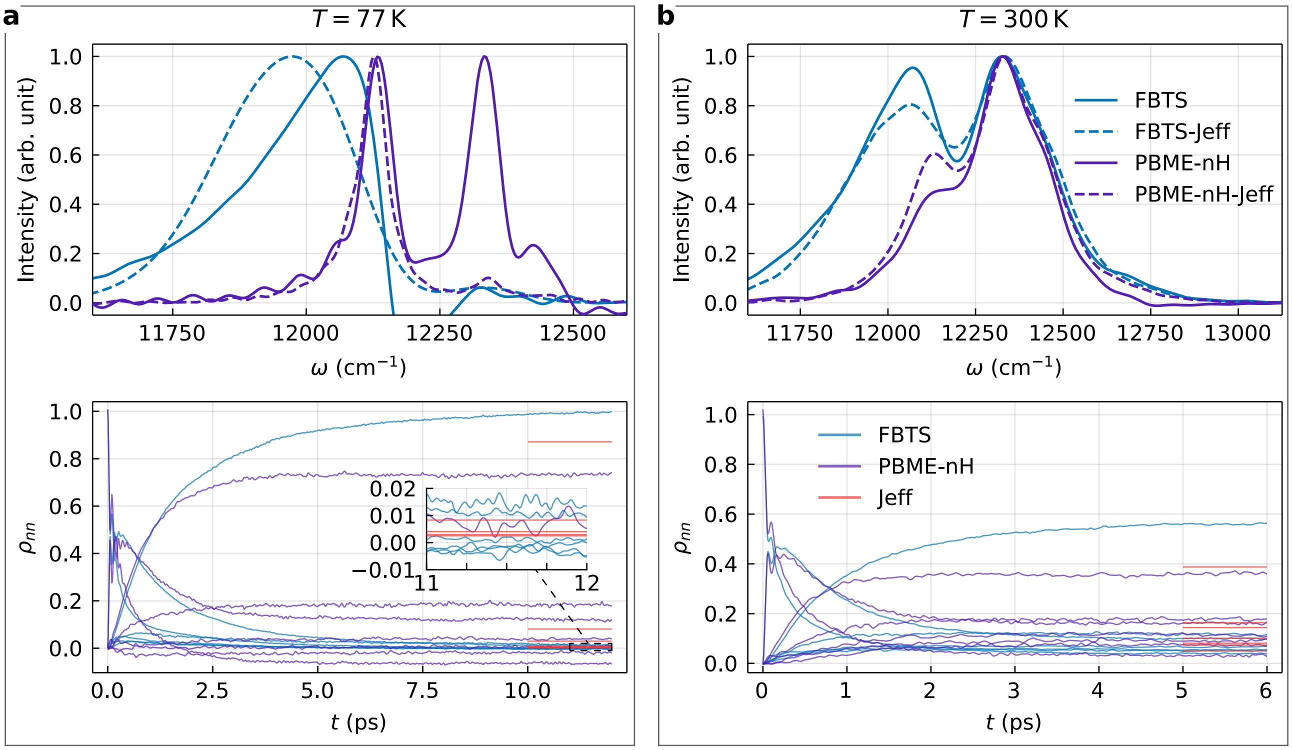


Figure S2. Fluorescence spectra of the 7 BChls FMO complex (upper plots) and population dynamics (lower plots) calculated using the B777 spectral density at **(a)** $T = 77$ K, **(b)** $T = 300$ K. The spectra are normalized to unit maximum intensity. The $y$-coordinates of the red horizontal lines indicate the equilibrium values as given by the effective-coupling theory.

Based on the above reasoning, we conclude that the PBME-nH-Jeff should be suitable for obtaining qualitatively correct results using a reasonable amount of computational resources.

* andrius.gelzinis@ff.vu.lt


[1] A. Gelzinis, L. Valkunas. Analytical derivation of equilibrium state for open quantum system. *J. Chem. Phys.* **2020**, *152*, 051103.

[2] *https://gitlab.com/jakovbraver/fbts*.

[3] J. Ma, J. Cao. Förster resonance energy transfer, absorption and emission spectra in multichromophoric systems. I. Full cumulant expansions and system-bath entanglement. *J. Chem. Phys.* **2015**, *142*, 094106.

[4] A. Gelzinis, D. Abramavicius, L. Valkunas. Absorption lineshapes of molecular aggregates revisited. *J. Chem. Phys.* **2015**, *142*, 154107.
